# Training Session Models in Endurance Sports: A Norwegian Perspective on Best Practice Recommendations

**DOI:** 10.1007/s40279-024-02067-4

**Published:** 2024-07-16

**Authors:** Espen Tønnessen, Øyvind Sandbakk, Silvana Bucher Sandbakk, Stephen Seiler, Thomas Haugen

**Affiliations:** 1 School of Health Sciences, Kristiania University College, PB 1190 Sentrum, 0107 Oslo, Norway; 2 Department of Neuromedicine and Movement Science, Centre for Elite Sports Research, Norwegian University of Science and Technology, 7491 Trondheim, Norway; 3 Department of Teacher Education, Norwegian University of Science and Technology, 7491 Trondheim, Norway; 4 Faculty of Health and Sport Sciences, University of Agder, PB 422, 4604 Kristiansand, Norway

## Abstract

**Background:**

Our scientific understanding of the mechanistic and practical connections between training session prescriptions, their execution by athletes, and adaptations over time in elite endurance sports remains limited. These connections are fundamental to the art and science of coaching.

**Objective:**

By using successful Norwegian endurance coaches as key informants, the aim of this study is to describe and compare best practice session models across different exercise intensities in Olympic endurance sports.

**Methods:**

Data collection was based on a four-step pragmatic qualitative study design, involving questionnaires, training logs from successful athletes, and in-depth and semi-structured interviews, followed by negotiation among researchers and coaches to assure our interpretations. Twelve successful and experienced male Norwegian coaches from biathlon, cross-country skiing, long-distance running, road cycling, rowing, speed skating, swimming, and triathlon were chosen as key informants. They had been responsible for the training of world-class endurance athletes who altogether have won > 370 medals in international championships.

**Results:**

The duration of low-intensity training (LIT) sessions ranges from 30 min to 7 h across sports, mainly due to modality-specific constraints and load tolerance considerations. Cross-training accounts for a considerable part of LIT sessions in several sports. Moderate (MIT)- and high-intensity training (HIT) sessions are mainly conducted as intervals in specific modalities, but competitions also account for a large proportion of annual HIT in most sports. Interval sessions are characterized by a high accumulated volume, a progressive increase in intensity throughout the session, and a controlled, rather than exhaustive, execution approach. A clear trend towards shorter intervals and lower work: rest ratio with increasing intensity was observed. Overall, the analyzed sports implement considerably more MIT than HIT sessions across the annual cycle.

**Conclusions:**

This study provides novel insights on quantitative and qualitative aspects of training session models across intensities employed by successful athletes in Olympic endurance sports. The interval training sessions revealed in this study are generally more voluminous, more controlled, and less exhaustive than most previous recommendations outlined in research literature.

## Key Points

**Table:** 

This study describes training session models across intensities applied by world-leading coaches in endurance sports.
Training session models vary substantially across sports, mainly due to load tolerance considerations for the locomotion modality, seasonal circumstances, and sport-specific demands.
The interval training session models outlined here are more voluminous, more controlled, and less exhaustive than recommendations from many published intervention studies.

## Introduction

Numerous studies published over the last ~ 25 years have quantified the training characteristics of elite endurance athletes, in which annual training volumes range from ~ 500 to ~ 1200 h per year, distributed across 300–600 training sessions [1–29]. This large variation among equally successful performers is mainly explained by modality-specific constraints (e.g., weight-bearing versus nonweight bearing sports, type of muscle action involved, cycle/muscle-contraction time, and leg-dominant versus whole-body exercise), although individual predispositions also matter [27]. Depending on the specific quantification approach (i.e., categorizing the distribution of whole sessions versus minute-for-minute time in zone), about 80–90% of endurance training is performed at low intensity (below the first lactate or ventilatory turn point), while the remaining 10–20% is performed at higher intensity [2, 7, 14, 18, 22]. While the interaction between training volume and intensity distribution is well described at an annual and monthly level in rowing [1–5], cross-country (XC) skiing [7, 8, 24–26], road cycling [9–12], long-distance running [13–16, 20–23], swimming [17–19], and triathlon [27–29], corresponding information for prescription and execution of individual endurance training sessions are sparse.

Training prescription for continuous exercise sessions include selection of exercise modality, working duration, and intensity, while interval training also encompasses manipulation of number of repetitions, number of series, relief interval intensity and duration, and the between-series recovery duration and intensity [31]. Ironically, interval training sessions are often described in more detail compared with continuous exercise training [2, 20, 31], although the latter training form by far constitutes the largest proportion of training in elite endurance athletes.

Session model comparisons across studies and sports are complicated because of inconsistent methodological frameworks (e.g., intensity zones) and terminology. Moreover, most of the research within this topic has been conducted on well-trained but nonelite volunteers who perform training sessions that may not be consistent with what “elite” endurance athletes perform [31]. Detailed training session descriptions have been presented for world-leading long-distance runners and XC skiers [20–26]. These studies show large between-sport differences in duration for low-intensity training (LIT), and partly also for moderate-intensity training (MIT) sessions, while the summated duration for high-intensity training (HIT) sessions appear more consistent. Corresponding training session descriptions for sports such as road cycling, swimming, triathlon, rowing, biathlon, and speed skating are clearly underrepresented or missing. Due to the large variations in annual training volume across endurance sports [30], it is reasonable to expect large variations in training session prescriptions as well.

Indeed, more research is needed to improve our understanding of session model features among elite endurance sports. The very best practitioners are often years ahead of sport science in integrating the critical features of training [2, 16, 22], and experienced coaches who have achieved success with multiple athletes over time are likely best capable to describe good session designs and identify factors ensuring high training consistency and quality [32, 33]. Surprisingly, the practices, knowledge and experience of the very best endurance sport coaches have received minimal attention in research literature.

Norway has been one of the world-leading sport nations per capita in the last two to three decades [34], with most Olympic and World Championships medals won in endurance sports such as XC skiing, biathlon, speed skating, rowing, cycling, swimming, long-distance running, and triathlon. One of the advantages of the Norwegian system is that endurance sports use the same framework for defining training content, facilitating valid comparisons across sports. By using successful Norwegian endurance coaches as key informants, the aim of this study is to describe and compare best practice session models across different training intensities in Olympic endurance sports. Within this context, we define endurance sports as disciplines with ≥ 6 min competition duration with an aerobic energy contribution of ≥  85%.

## Methods

### Study Design

This study is a part of a larger project investigating successful coaches in Olympic endurance sports, where the overall aim is to gain comprehensive insights regarding the holistic training philosophies and practices at the macro-, meso- and micro-level. For the current study, a pragmatic multiple case study design was used to investigate best practice session models successfully used to attain world-class performance in Olympic endurance sports. To investigate the complexity and capture sport-specific dimensions and perspectives, the following cases were selected: XC skiing, biathlon, swimming, long-distance running, long-track speed skating (hereafter referred to as speed skating), rowing, road cycling, and triathlon. To allow for comparison and contrast across sports, all cases were selected within Norway, assuming similar culture and context. Some of the most successful and experienced coaches were chosen as key informants.

### Participants

Twelve male Norwegian coaches participated in this study. They were all currently or previously responsible for the training of world-class endurance athletes who altogether have won more than 370 Olympic, World, and European Championship medals, mainly with Norwegian athletes. All coaches had experience of coaching both males and females. Two coaches were involved in XC skiing, biathlon, swimming, triathlon, and long-distance running, while one coach was involved in speed skating, rowing, and road cycling. One informant coached both swimming and triathlon athletes. Annual training volume measures prescribed by the coaches in these sports are presented in Table 1. All the coaches provided a written informed consent to participate prior to the study and approved the manuscript prior to submission. The Regional Committee for Medical and Health Research Ethics waived the requirement for ethical approval for this study and the ethics of the project was performed according to the institutional requirements at the School of Health Sciences, Kristiania University College. Approval for data security and handling was obtained from the Norwegian Centre for Research Data (reference no. 605672).

**Table 1:** Annual training volume measures (range) across the analyzed endurance sports

Sport	Hours per year	Sessions per year	Competition days per year	Intensive training days per year	% specific training
Biathlon	800–1000	500–575	30–40	100–120	> 60
Cross-country skiing	900–1100	525–575	30–40	100–120	> 60
Long-distance running	600–700	550–625	20–35	110–140	> 90
Road cycling	1000–1200	300–350	50–80	110–130	> 90
Rowing	850–1000	475–525	25–35	100–125	> 60
Speed skating	900–1100	500–575	25–35	120–140	> 15
Swimming	1150–1350	650–700	20–30	130–150	> 70
Triathlon	1200–1400	700–800	15–25	130–150	100

Swimming, cycling, and running in triathlon account for approximately 20–25, 25–30, and 45–50% of the annual training hours, respectively

### Procedures

Inspired by the key informant technique in ethnographic research, a pragmatic four-step procedure was used to collect and quality-assure comprehensive information on best practice regarding key session models across different endurance sports:

1. Initially, an extensive questionnaire related to planning, conducting, and evaluation of training at the macro, meso, micro, and session level was administered to all coaches.
2. The next step consisted of quality-assurance of data through conversations with the coaches and cross-referencing with historically reported training logs from some of their most successful athletes.
3. Thereafter, a semistructured interview was conducted with each coach by the first and second authors to obtain supplementary information related to the qualitative aspects of session models among elite endurance sports. Within this context, training quality is defined as the degree of excellence related to how the training process or training sessions are executed to optimize adaptations and/or improve overall performance [33]. Each interview lasted approximately 180 min, of which about one-third was directly related to this study. The interviews were audio recorded and transcribed. Formal translation and back translation from Norwegian to English were performed by the first and third author, respectively.
4. During analysis, we involved the coaches in an extensive review process and follow-up interviews to clarify and ensure that the findings reflected their perspectives on best practice training sessions as accurately as possible.

In terms of endurance training intensity quantification, a six-zone scale developed by the Norwegian Top Sport Centre (Table 2) was applied. Modified versions of this scale have been used in several previous studies [35–37]. Training zone determination during practice was determined by the coaches based on a holistic interpretation of all the included metrics listed in Table 2. Moreover, a modified session goal approach based on Sylta et al. [36] was employed. That is, training intensity distribution was described in terms of the categorical distribution of prescribed training sessions across intensity zones based on their execution. In comparison with a time-in-zone approach that will overemphasize LIT, this method presents a more representative picture of MIT and LIT prescription within long-term programming. Here, only the main part of the session was considered, while warmup and cool down were excluded. We use the term “accumulated work duration” (AWD) for interval sessions, and this is defined as the summated duration of the work bouts only.

**Table 2:** Intensity scale for elite endurance athletes

Scale		Heart rate	VO_2_	BLa	RPE_Borg_
6-zone	3-zone	(% max)	(% max)	(mmol/L)	6–20
6	HIT	NA	NA	> 10	18–20
5	HIT	> 93	94–100	6.0–10.0	18–19
4	HIT	88–92	88–93	4.0–6.0	17–18
3	MIT	83–87	81–87	2.5–4.0	15–16
2	LIT	73–82	66–80	1.5–2.5	13–14
1	LIT	60–72	50–65	< 1.5	10–12

*BLa* typical blood lactate (normative blood lactate concentration ranges based on red-cell lysed blood), *RPE* rating of perceived exertion (based on Borg’s 6–20 scale), *HIT* high intensity training, *MIT* moderate intensity training, *LIT* low intensity training

For the purpose of this study, cross training was defined as endurance training in a nonspecific mode. Treadmill running (including antigravity treadmill running), roller skiing, roller skating, ergometer rowing, and indoor cycling were considered specific (i.e., not cross training) for runners, cross-country skiers/biathletes, speed skaters, rowers, and cyclists, respectively.

### Analyses

Numerical information on training session organization across intensity zones was systematized in Microsoft Excel (Microsoft Corporation, Redmond, WA) for descriptive presentations. Thereafter, this information was compared with information from training diaries of successful athletes in the respective sports and calibrated among the authors and coaches.

To identify similarities and differences within and across endurance sports, summaries of common session-model features across endurance sports and sport-specific features related to planning and execution of training sessions were outlined.

## Results

Table 2 presents commonly applied training session models across intensity zones among Norwegian world-leading athletes in Olympic endurance sports, while Table 3 provides an overview of how the loading factors are typically organized across intensity zones within the same sports. Overall, LIT sessions account for approximately 75–80% of all sessions. These are dominated by continuous exercise, although swimming, rowing, speed skating, and road cycling also apply low-intensive intervals. Athletes from all disciplines surveyed performed the vast majority of LIT sessions in Z1 and only to a limited extent in Z2. The duration of typical continuous LIT sessions spans from 30 to 420 min across sports, with long-distance running on the lower end and road cycling on the upper end of the scale. Long-distance running, road cycling, swimming and triathlon perform most LIT sessions in the specific modalities, while nonspecific cross-training accounts for a considerable proportion of total LIT sessions in speed skating, rowing, biathlon, and XC skiing.

**Table 3:** Commonly applied training session models across intensity zones in Norwegian world-leading endurance athletes

Sport	Z1	Z2	Z3	Z4	Z5	Z6/7
Biathlon Example 1 Example 2 Example 3	(4–7 weekly sessions) Continuous: 1:30–2:30 h ski skating Continuous: 1:00–2:00 h ski double poling Continuous: 2:30–4:00 h cycling or running in soft terrain	“Pure” Z2 sessions are rarely applied, but long-slow-distance sessions may include 15–45 min Z2 work to maintain effective technique uphill	(1–3 weekly sessions) Intervals: 5–8 × 8 min uphill ski skating, *R* = 2 min Continuous: 45–60 min skiing or roller ski skating Intervals: 4 × 15 min mountain-bike cycling, *R* = 2–3 min	(0–2 weekly sessions) Intervals: 5–8 × 4–5 min ski skating, *R* = 2–3 min Intervals: 5–6 × 6 min uphill running w/w.o. poles, *R* = 2–3 min Continuous: 7.5–15 km (20–45 min) ski test race	(0–2 weekly sessions) Intervals: 4–6 × 2–4 min ski skating, *R* = 2–4 min Intervals: 6 × 4 min uphill running w/w.o. poles, *R* = 2–4 min Intervals: 5–8 × 2–3 min ski skating, *R* = 1–2 min	(0–1 weekly sessions) Intervals: 6–10 × 20–30 s ski skating, *R* = 1–2 min Intervals: finish sprints on certain Z5 sessions
XC skiing Example 1 Example 2 Example 3	(4–7 weekly sessions) Continuous: 1:30–2:00 h running or skiing Continuous: 2–3 h skiing or running in soft terrain Continuous: 3–4 h running in soft terrain	“Pure” Z2 sessions are rarely applied, but long-slow-distance sessions may include 15–45 min Z2 work to maintain effective technique uphill	(1–3 weekly sessions) Intervals: 5–8 × 8 min running or skiing, *R* = 2 min Continuous: 45–60 min skiing Intervals: 4 × 15 min skiing, *R* = 2 min	(0–2 weekly sessions) Intervals: 6–8 × 4–5 min uphill running w/w.o. poles, *R* = 2–3 min Intervals: 5–6 × 5–6 min uphill skiing, *R* = 2–3 min Continuous: 10–15 km (25–40 min) ski test race	(0–2 weekly sessions) Intervals: 5–6 × 3–5 min skiing, *R* = 2–3 min Intervals: 8–10 × 2 min skiing or uphill running w/poles, *R* = 1 min	(0–1 weekly sessions) Intervals: 8–12 × 1 min skiing, *R* = 1 min Intervals: 4–5 × 2–3 min skiing, *R* = 5–10 min
LD running Example 1 Example 2 Example 3	(5–9 weekly sessions) Continuous: 8–14 km (30–50 min) running Continuous: 14–17 km (50–75 min) running Continuous: 20–25 km (1:15–1:45 h) running	“Pure” Z2 sessions are rarely applied, but progressive long runs may include 5–30 min running in Z2	(2–5 weekly sessions) Intervals: 10–12 × 1000 m (~ 3:00 min) running, *R* = 45–60 s Intervals: 6 × 5 min running, *R* = 1 min Intervals: 3 × 3 km (9–10 min) running, *R* = 2 min	(0–2 weekly sessions) Intervals: 6–8 × 1000 m (2:45–3:00 min) running, *R* = 1:30 min Intervals: 20–25 × 400 m (65–70 s) running, *R* = 30–45 s Intervals: 4–6 × 2 km (6:00–6:20 min) running, *R* = 2 min	(0–1 weekly sessions) Intervals: 4–6 × 1000 m (2:30–3:00 min) running, *R* = 2 min Intervals: 10–15 × 400 m (60–70 s) running, *R* = 60–75 s Intervals: 20 × 200 m (30–40 s) hill repeats, *R* = easy jog back (~ 60 s)	(0–1 weekly sessions) Intervals: 5 × 300 m (35–40 s) running, *R* = 3–5 min Intervals: 10–12 × 200 m (25–30 s) running, *R* = 30–60 s
Road cycling Example 1 Example 2 Example 3	(3–6 weekly sessions) Continuous: 3–7 h cycling (small parts in Z2 due to terrain variations) Continuous: 1–2 h low-gear cycling (after competition or HIT) Continuous: great parts of competition races are performed in Z1 (depending on terrain, role in the team, etc.)	(1–2 weekly sessions) Continuous: parts of easy long rides or competitions occur in Z2, depending on terrain and role within the team Intervals: 8–10 × 3–6 min low-cadence (RPM 40–60) cycling with one or two legs, *R* = 1–2 min easy pedaling	(1–3 weekly sessions) Intervals: 4 × 10–15 min cycling, *R* = 2 min. Or 6 × 8 min, *R* = 1–2 min Continuous: 1 h cycling Competitions: long-tempo stages and mountain climbs in long tour stages mainly occur in Z3	(0–1 weekly sessions) Intervals: 4–8 min reps for a total of 45 min, 2–3 min easy cycling in between Intervals: 2–4–6–8–10–8–6–4–2 min, *R* = 1–3 min Intervals: motor-paced interval cycling (Z2–5), ~ 1 h total session duration (10–20 min in Z4)	(0–1 weekly sessions) Intervals: 10 × 1–3 min, *R* = 1 min Intervals: 15–25 min of short intervals with varying work durations (15–60 s) and brief recoveries (15–30 s)	(0–1 weekly sessions) Intervals: 4–5 × 30–90 s all-out repeats, *R* = 5 min Intervals: specific sprint-training with gradually increased speed while drafting behind team-mates before a 15 s full sprint
Rowing Example 1 Example 2 Example 3	(5–8 weekly sessions) Continuous: 1–2 h (12–20 km) rowing Continuous: 2:30–3:00 h (26–30 km) rowing Continuous: 2–4 h skiing or cycling	(1–2 weekly sessions) Intervals: 3–4 × 20 min rowing, *R* = 2 min Intervals: 4 × 15 min rowing, *R* = 2–5 min Continuous: 60 min rowing	(1–2 weekly sessions) Intervals: 3–4 × 15–20 min rowing, *R* = 4 min Intervals: 6–8 × 10 min rowing, *R* = 2–3 min Intervals: 6 × 6 min uphill running, *R* = 2 min	(0–1 weekly sessions) Intervals: 4–5 × 10 min rowing, *R* = 4–5 min Intervals: 8–12 × 4 min rowing, *R* = 1–2 min Intervals: 4–6 × 6–8 min rowing, *R* = 2–3 min	(0–1 weekly sessions) Intervals: 6–8 × 5 min rowing, *R* = 2–3 min Intervals: 5–7 × 4 min rowing, *R* = 2–3 min Intervals: 4–5 × 7 min rowing, *R* = 3–5 min	(0–1 weekly sessions) Intervals: 5–7 × 500 m rowing (~ 1:30 min) rowing, *R* = 5 min Intervals: 2–3 × 1000 m rowing (~ 3 min), *R* = 5–7 min
Speed skating Example 1 Example 2 Example 3	(4–7 weekly sessions) Continuous: 2–5 h road cycling Intervals: 3–5 × 20–30 min roller skating outdoors, *R* = 1–3 min Continuous: 1–2 h on bike trainer (after skating sessions)	(1–3 weekly sessions) Intervals: 3 × 10 min + 4 × 5 min inline/roller skating, *R* = 3 min Intervals: 5 × 10–12 min roller skating, *R* = 5 min Intervals: 4 × 12 min inline/roller skating, *R* = 3 min	(1–3 weekly sessions) Intervals: 6 × 8 min road cycling on flat or uphill, *R* = 2 min Intervals: 4–5 × 15 min uphill road cycling, *R* = ~ 6 min (roll back) (Specific speed skating rarely applied in Z3)	(0–1 weekly sessions) Intervals: 4 × 5000 m (6–7 min) skating, *R* = 5–10 min Intervals: 4 × 6–10 min progressive skating, *R* = 3 min Intervals: 10 × 3–4 min skating, *R* = 3 min	(0–2 weekly sessions) Intervals: 8–10 × 1600 or 2000 m (2:15–3:00 min) skating, R = 3–7 min Intervals: 4 × 5000 m (~ 6–7 min) skating, R = 5–10 min Intervals: 4 × 12 min progressive skating (last 2–4 min of each interval in Z5), R = 4 min	(0–1 weekly sessions) Intervals: 2 × (8–10 × 500 m (~ 40 s) skating, *R* = 700 m sliding (~ 1:30 min), SR = 5–7 min Intervals: 3 × 1600 m (~ 1:50–2:00 min) team pursuit skating, *R* = 5–8 min, SR = 10–15 min. (2 × 10 min Z4 skating afterwards)
Swimming Example 1 Example 2 Example 3	(7–9 weekly sessions) Intervals: 3–6 × 1500 m (17–19 min) crawl, *R* = 30–60 s Intervals: 5–10 × 800 m (9:15–10:30 min) crawl, *R* = 30–45 s Intervals: 2–3 × 800 m (9:15–10:30 min) + 2 × 400 m (4:40–5:00 min) + 4 × 200 m (2:20–2:30 min) crawl, *R* = 15–30 s	(1–2 weekly sessions) Intervals: 5–8 × 800 m (8:45–9:45 min crawl, *R* = 30–60 s Intervals: 6–10 × 600 m (6:30–7:15 min) crawl, *R* = 30–60 s Intervals: 4–6 × [5 × 100 m (4:15–4:30 min) + 1 × 500 m (5:30–6:15 min)], R = 15–30 s	(1–3 weekly sessions) Continuous: 4000 m (43–45 min) crawl Intervals: 4–6 × 1000 m (11:00–12:00 min) crawl, *R* = 1:30–2:00 min Intervals: 20–24 × 200 m (2:05–2:20 min) crawl, *R* = ~ 30 s	(0–1 weekly sessions) Intervals: 4 × 800 m crawl (8:30–9:30 min), *R* = 2:30 min Intervals: 6–8 × 400 m (4:10–4:40 min) crawl, *R* = 60 s Intervals: 12–15 × 200 m (2:00–2:10 min crawl, *R* = 60 s	(0–1 weekly sessions) Intervals: 3–5 × 400 m (3:55–4:25 min), R = 2–3 min Intervals: 4–6 × 300 m (2:55–3:15 min) crawl, R = 2–3 min Intervals: 8–10 × 200 m (1:55–2:05 min crawl, R = 60–90 s	(0–1 weekly sessions) Intervals: 8–16 × 50 m (25–30 s) crawl, *R* = 1:00–1:30 min Intervals: 8 × 50 m (27–30 s) crawl, *R* = 10–15 s Intervals: 2–3 × (5 × 100 m (57–63 s), *R* = 10–20 s, 200 “gliding” crawl between sets
Triathlon Example 1 (swimming) Example 2 (road cycling) Example 3 (running) Brick workouts (combined sessions)	(7–11 weekly sessions) “Pure” Z1 swimming sessions are rarely applied, but warmup and cool down may occur in Z1 Continuous: 2:00–3:30 h road cycling Continuous: 1–2 h running NA	(2–3 weekly sessions) Intervals: 5 × 800 m (9:30–11 min) crawl, *R* = 45–60 s. Or 18–20 × 200 m (2:25–2:45 min) crawl, *R* = 20–30 s Continuous: 1–2 h road cycling Continuous: 45–90 min running NA	(3–6 weekly sessions) Intervals: 30–35 × 100 m crawl (1:08–1:20 min), *R* = 30 s. Or 8 × 400 m (4:40–5:15 min) crawl, *R* = 1 min Intervals: 8–11 × 8 min cycling, *R* = 1–2 min Intervals: 10–14 × 1000 m (3:00–3:30 min) running, *R* = 1 min Brick 1: 8–10 × 200 m (2:10–2:30 min) crawl, *R* = 30 s. Then 60 min continuous road cycling with inlaid accelerations (600–1000 W) Brick 2: 50–60 min road cycling followed by 8–10 km (25–40 min) running	(1–2 weekly sessions) Intervals: 40–60 × 50 m (29–35 s) crawl, *R* = 15 s. Or 6 × 400 (4:30–5:00 min) crawl, *R* = 1:30 min NA NA Brick 1: 15–20 × 100 m (1:05–1:15 min) crawl, *R* = 15–20 s. Then 50–60 min continuous road cycling with inlaid accelerations 600–1000 W) every third min Brick 2: 50–60 min cycling followed by 5 × 2 km (5:50–6:40 min) running intervals (*R* = 1 min)	(0–1 weekly sessions) Intervals: 15–20 × 100 m (1:01–1:12 min) crawl, R = 1:30 min Intervals: 6–8 × 3 min road cycling, R = 2 min Intervals: 6 × 1000 m (2:50–3:20 min) running, R = 2 min NA	(0–1 weekly sessions) Intervals: 16 × 50 m (27–33 s) crawl, *R* = 60 s. Or 8 × 100 m (0:57–1:10 min) crawl, *R* = 90 s NA NA NA

The most typical sessions are listed first (example 1), while the other sessions (example 2 and 3) are applied less frequently. Upper range values for duration/distance, sets and repetitions reflect typical preparation-period sessions, while corresponding lower range values reflect competition-period sessions. Upper and lower range for interval times denote women and men, respectively

*Z* intensity zone,* LD* long distance* XC* cross country, *R* recoveries between repetitions (and sets where stated),* w/w.o.* with or without. “Pure” Z2 sessions are rarely applied in long-distance running, XC skiing, and biathlon. In rowing, swimming and speed skating, Z2 training is performed for technical development purposes. Warmup and cool down are performed in addition to the described interval training sessions. Typical warmup routines for runners, road cyclists, XC skiers, biathletes, and triathletes include 20–30 min in a specific modality where the intensity progresses gradually from Z1 to Z3 at the latter part (runners and cyclists also perform three to five strides at the end of the warmup). Speed skaters perform 20–30 min cycling in Z1–2, followed by 10–20 min skating imitation exercises, four to six skating rounds on track with progressive intensity (Z1–4), and 2–4 × 30–40 s skating in Z5–6. Swimmers typically perform 15–30 min of land-based mobility exercises with and without rubber bands, 15–20 min progressive swimming in Z1–3, and, finally, 3–5 × 5–10 s swimming sprints. Rowers typically perform 20–30 min ergometer cycling or rowing in Z1–2 and 10–20 min rowing on water in Z1–3, followed by two to three repetitions of accelerated rowing sprints. Cool downs for most sports typically consist of 20–30 min Z2–1 (i.e., regressive work) in a specific modality, except for speed skaters and rowers, who perform 30–60 min cycling in Z1

In XC skiing, most athletes train around 50% in the skating and 50% in the classic style. All ski sessions for XC and biathlon can also be performed on roller skies. In biathlon, all Z3-4 sessions can include shooting during the rest intervals. Specific rowing sessions can be performed on a rowing ergometer. Similarly, specific speed-skating sessions can be performed on roller skates. The running sessions for triathletes can be performed on paved/dirt roads or rubberized track

MIT sessions (i.e., Z3) account for approximately 10–15% of all sessions across the annual cycle. These are mainly performed as intervals, although with large sport-specific variations. AWD is in the range of 20–90 min, and interval times are mainly in the range 5–20 min, while work-to-rest ratio is mainly in the range 6–4:1 (Tables 3 and 4). Biathlon, XC skiing, road cycling, and swimming also apply continuous work in Z3, with accumulated work duration in the range 40–60 min. In several sports, particularly road cycling, competitions account for a considerable part of the overall Z3 volume.

**Table 4:** Loading factor organization in typical training sessions across intensity zones

Sports and loading factors	Z1	Z2	Z3	Z4	Z5	Z6-7
**Biathlon**						
AWD (min per session)	60–240	15-45^a^	30–65	20–40	15–25	5–10
% specific/nonspecific modality	> 70/< 30	> 80/< 20	> 70/< 30	> 80/< 20	> 80/< 20	100/NA
Method	Cont.	Cont.	Cont. or int.	Int. or comp.	Int. or comp.	Int
Work interval duration (min)	NA	NA	8–15	2–6	0:30–4	0:20–1
Total number of intervals per session	NA	NA	4–8	5–8	4–8	6–10
Recoveries (min) between reps (sets)	NA	NA	2–3 (NA)	2–3 (NA)	0:15–4 (NA)	1–3 (NA)
Work-to-rest ratio			7–3:1	3–2:1	2–1:1	0.5–0.25:1
Passive or active recoveries			Both	Both	Passive	Passive
**XC skiing**						
AWD (min per session)	60–240	15-45^a^	40–65	20–40	15–25	5–12
% specific/nonspecific modality	> 70/< 30	> 70/< 30	> 70/< 30	> 70/< 30	> 70/< 30	> 80/< 20
Method	Cont.	Cont.	Cont. or int.	Int. or comp.	Int. or comp.	Int
Work interval duration (min)	NA	NA	8–15	2–6	0:30–5	0:20–3
Total number of intervals per session	NA	NA	4–8	5–8	5–10	4–12
Recoveries (min) between reps (sets)	NA	NA	~ 2 (NA)	2–3 (NA)	0:15–3 (NA)	1–10 (NA)
Work-to-rest ratio			7–4:1	3–2:1	1.5–1:1	1–0.2:1
Passive or active recoveries			Both	Both	Passive	Passive
**LD running**						
AWD (min per session)	30–105	5-30^a^	20–40	15–35	10–20	3–6
% specific/nonspecific modality	> 90/< 10	100	100	100	100	100
Method	Cont	Cont	Cont. or int.	Int. or comp.	Int. or comp.	Int
Work interval duration (min)	NA	NA	3–12	1–6	0:30–3	0:20–0:40
Total number of intervals per session	NA	NA	5–12	4–25	4–20	5–12
Recoveries (min) between reps (sets)	NA	NA	1–2 (NA)	0:30–3 (NA)	1–2 (NA)	0:30–5 (NA)
Work-to-rest ratio			5–3:1	2–1.5:1	1.5–0.5:1	1–0.1:1
Passive or active recoveries			Both	Passive	Both	Passive
**Road cycling**						
AWD (min per session)	120–420	20–60	45–60	20–50	10–30	4–8
% specific/nonspecific modality	100/NA	100/NA	100/NA	100/NA	100/NA	100/NA
Method	Cont	Cont. or int.	Int. or cont	Int	Int	Int
Work interval duration (min)	NA	3–6	8–15	2–10	0:15–3	0:20–1:30
Total number of intervals per session	NA	8–10	4–6	4–10	4–10	4–10
Recoveries (min) between reps (sets)	NA	1–2	1–2	1–3	0:15–1	3–5
Work-to-rest ratio		10–4:1	10–4:1	4–2:1	3–1:1	0.3–0.1:1
Passive or active recoveries		Both	Both	Both	Both	Both
**Rowing**						
AWD (min per session)	60–240	60–80	45–80	30–50	20–40	7–10
% specific/nonspecific modality	> 70/ < 30	100/NA	100/NA	100/NA	100/NA	100/NA
Method	Cont	Cont. or int.	Int	Int	Int. or comp.	Int
Work interval duration (min)	NA	15–20	6–20	4–10	4–7	1:30–3
Total number of intervals per session	NA	3–4	3–8	4–12	4–8	2–7
Recoveries (min) between reps (sets)	NA	2–5 (NA)	2–4 (NA)	1–5 (NA)	2–5 (NA)	5–7 (NA)
Work-to-rest ratio		10–4:1	5–3:1	4–2:1	2.5–1.5:1	0.6–0.3:1
Passive or active recoveries		Both	Both	Passive	Passive	Passive
**Speed skating**						
AWD (min per session)	60–300	50–60	45–75	25–40	20–30	6–12
% specific/nonspecific modality	< 10/ > 90	> 70/ < 30	< 10/ > 90	100/NA	100/NA	100/NA
Method	Cont. or int.	Int	Int	Int	Int. or comp.	Int. or comp.
Work interval duration (min)	15–30	10–15	8–15	3–10	2–7	0:40–2
Total number of intervals per session	3–5	3–7	4–6	4–10	4–10	3–20
Recoveries (min) between reps (sets)	1–3 (NA)	3–5 (NA)	2–6 (NA)	3–10 (NA)	3–10 (NA)	1:30–8:00 (NA)
Work-to-rest ratio	20–10:1	5–1.5:1	5–3:1	3–0.7:1	0.5–1:1	0.4–0.3:1
Passive or active recoveries	Passive	Both	Active	Both	Both	Both
**Swimming**						
AWD (min per session)	60–150	40–80	45–70	25–40	12–20	4–15
% specific/nonspecific modality	> 90/ < 10	100/NA	> 90/ < 10	100/NA	100/NA	100/NA
Method	Int. or cont	Int	Int. or cont	Int	Int. or comp.	Int
Work interval duration (min)	2–20	4–10	2–12	2–10	1–5	0:30–1
Total number of intervals per session	3–10	5–10	4–25	4–15	3–10	8–16
Recoveries (min) between reps (sets)	0:15–1 (NA)	0:15–1 (NA)	0:30–2 (NA)	1:00–2:30 (NA)	1–3 (NA)	0:10–1:30 (NA)
Work-to-rest ratio	50–20:1	20–7:1	7–4:1	4–2:1	2–1:1	7–0.3:1
Passive or active recoveries	Passive	Passive	Both	Both	Both	Both
**Triathlon—swimming**						
AWD (min per session)	NA^b^	45–60	30–45	20–30	15–22	NA
% specific/nonspecific modality	NA	100/NA	100/NA	100/NA	100/NA	NA
Method	NA	Int	Int	Int. or comp.	Int	NA
Work interval duration (min)	NA	2:30–12	1–5	0:30–5	0:30–1:30	NA
Total number of intervals per session	NA	5–20	8–35	6–60	10–20	NA
Recoveries (min) between reps (sets)	NA	0:20–1 (NA)	0:30–1 (NA)	0:15–1:30 (NA)	1–2/(NA)	NA
Work-to-rest ratio		10–5:1	5–2.5:1	3–2:1	1.3–0.7:1	
Passive or active recoveries		Passive	Passive	Passive	Both	
**Triathlon—cycling**						
AWD (min per session)	120–210	60–120	60–90	NA^3^	15–25	NA
% specific/nonspecific modality	100/NA	100/NA	100/NA	NA	100/NA	NA
Method	Cont	Cont	Int. and comp	NA	Int	NA
Work Interval duration (min)	NA	NA	8–10	NA	2–4	NA
Total number of intervals per session	NA	NA	6–11	NA	6–12	NA
Recoveries (min) between reps (sets)	NA	NA	1–2 (NA)	NA	0:15–2	NA
Work-to-rest ratio			7–4:1		2–1.3:1	
Passive or active recoveries			Both		Passive	NA
**Triathlon—running**						
AWD (min·session^−1^)	60–120	45–90	30–50	NA^c^	15–20	NA
% specific/non-specific modality	100/NA	100/NA	100/NA	NA	100/NA	NA
Method	Cont	Cont	Int. or comp.	NA	Int	NA
Work Interval duration (min)	NA	NA	3–7	NA	2–4	NA
Total number of intervals per session	NA	NA	5–14	NA	4–8	NA
Recoveries (min) between reps (sets)	NA	NA	1–1:30 (NA)	NA	1–3 (NA)	NA
Work-to-rest ratio			5–3:1		2–1.3:1	
Passive or active recoveries			Passive		Passive	
**Triathlon—brick workouts**						
AWD (min·session^−1^)	NA	NA	75–100	60–80	NA	NA
% specific/non-specific modality	NA	NA	100/NA	100/NA	NA	NA
Method	NA	NA	Int. and/or cont.	Int. and/or cont.	NA	NA
Work Interval duration (min)	NA	NA	1–7	1–3	NA	NA
Total number of intervals per session	NA	NA	5–20	5–20	NA	NA
Recoveries (min) between reps (sets)	NA	NA	0:15–1	0:15–1	NA	NA
Work-to-rest ratio			7–4:1	4–3:1		
Passive or active recoveries			Passive	Passive		

*Z* intensity zone, *AWD* typical accumulated work duration, *cont.* continuous work, *int.* intervals, *comp.* competitions, *R* recoveries between repetitions (and sets where stated), *NA* not applied

^a^“Pure” Z2 sessions are rarely applied, but parts of long slow distance may include Z2 work for technical purposes or terrain variations

^b^“Pure” Z1 swimming sessions are rarely applied by triathletes, but warmups and cool downs may include Z1 work

^c^“Pure” Z4 cycling or running sessions are rarely applied by triathletes, but these modalities are included in Z4 brick sessions. Note that the loading factor organization presented here does not include training sessions during tapering periods or easy training weeks. Moreover, the number of intervals can vary considerably within and between sports and intensity zones. Long-interval sessions typically consist of three to six repetitions, while short-interval sessions consist of 15–25 repetitions

HIT sessions comprise about 5–10% of all sessions and are mainly conducted as intervals and competitions in all sports. AWD is in the range 15–50 min for Z4, 10–30 min for Z5, and 3–15 min for Z6/7 (warmup and cool down not included), while work bout durations are in the range 1–10 min, from 30 s to 7 min, and from 20 s to 3 min, respectively (Table 3 and 4). Work-to-rest ratio is in the range 3–2:1 for Z4, 2–1:1 for Z5, and 1–0.1:1 for Z6/7, but sport-specific differences are clearly present. Competitions account for a considerable part of Z4/5 work among elite athletes, as most sports have at least 15–20 competition days per year (Table 1).

Most coaches reported applying a limited set of session models within each zone for week-to-week calibration/control of performance development. Within these sessions, several common features were identified across endurance sports that are described in detail in Table 5. These include the application of hard–easy rhythmicity, few but well-known session models, lactate measurements for intensity control, limited use of all-out endurance training sessions, mixing intensity zones within sessions, slight progressive intensity increases throughout the hard session(s), adjustments of session models during altitude training, and a preference towards passive instead of active recoveries during interval sessions. Moreover, some common characteristics related to the coaches’ focus before, during, and after the sessions are also present (Table 5).

**Table 5:** Common session-model features across endurance sports

Common features	Descriptions
Hard–easy rhythmicity	Days of hard workouts (i.e., interval training or extra-long slow-distance sessions) are systematically alternated with days of easy low-intensity training in between. Most coaches advocate two to three hard training days (so called key sessions) per week during the preparation period (e.g., Tuesdays, Thursdays, and Saturdays)
Double intensive sessions	A total of 7 out of the 13 interviewed coaches practice double intensive sessions (i.e., intervals both in the morning and afternoon session of the same day). The main purpose is to increase the total volume of intensive training while managing recovery cycles and stress load. In long-distance running, swimming, and triathlon, this approach is applied in Z3 sessions. Rowing and speed skating apply double intensive sessions in Z4–6 to increase the amount of training around race pace
Cross-training	Most sports apply cross-training (mainly in Z1) to achieve sufficient total training volume, although in varying degrees. Training sessions in Z3–5 are mainly conducted in a specific modality, except for speed skating, XC skiing, and biathlon, who perform cross-training systematically also at these intensities (only Z3 for speed skating). A common notion among the coaches is that cross-training modality must bear sufficient physiological and mechanical resemblances to the specific demands to maximize the odds for positive adaptations
Few session models	Most coaches apply a limited set of session models within each zone for predictability and week-to-week calibration/control purposes
Mostly controlled, very few “all-out” sessions	Very few hard sessions (competitions not included) across the annual cycle are conducted to complete exhaustion, but rather with a “reps in reserve” approach. The main purpose with this approach is to increase the accumulated working volume at high (but not too high) intensities and ensure that the athletes are sufficiently recovered for the next key session. All-out sessions (which are very similar to the competition-specific demands) are only performed the last 3–6 weeks prior to the main competition of the macrocycle. Elite coaches seek sustainability and optimization through session programming, not maximization
Progressive intensity increases throughout the session(s)	Most hard sessions are performed with a slight progressive increase in intensity. The difference between the first and last interval may be 0.5 km/h during running intervals and 10–25 W during cycling intervals. Similarly, continuous long-slow distance sessions typically start at the lower end of the intensity zone, then gradually increase to the mid or upper end of the zone as the session progresses
Combination of intensity zones	Table 1 presents the most used sessions models within each intensity zone. However, the coaches also implement sessions that combine training intensities. Combinations of Z1/2, Z3/4, and Z4/5 are most often applied
Altitude training	Most coaches advocate altitude training. Altitude sessions are conducted with lower speed/power output compared to sea-level sessions, leading to lower neuromuscular loading. During altitude interval sessions (mainly Z3–4), the recovery periods tend to be somewhat longer than corresponding sessions at sea level to avoid accumulation of fatigue and keep the intensity at the desired level
Tapering strategies and easy weeks	During tapering or easy training weeks, about 50% shorter session duration than those presented in Table 1 are advocated. The main intention with such sessions is to decrease the cumulative effects of fatigue while maintaining fitness/capacity
Passive recoveries	Most coaches apply passive recoveries between intervals. Active recoveries are mainly used for training organization/logistic purposes
Coach’s focus prior to the session(s)	The coaches spend considerable time on planning optimal sessions, often in cooperation with the athletes. The training content of key sessions is typically presented 1–7 days in advance to facilitate athletes’ mental preparations
Coach’s focus during the sessions(s)	The main focus during interval sessions is to provide technical feedback/guidance, modify training load variables when necessary, and assist the athletes with intensity control (lactate samples, timing/power output assessments, etc.). Similar focus is present on low-intensive sessions, although less frequent measurements for intensity control purposes are applied. Lactate samples are considered more important than speed/power output for developing the athletes’ inner feeling of intensity
Coach’s focus after the session(s)	Most coaches practice debriefing and recapitulation of each session together with the athlete(s) to pinpoint what worked well and features for improvements. The main intention is to create an arena for learning and enhance training quality for subsequent sessions

Although several consistent approaches were observed in terms of training session organization and implementation, some sport-specific features were identified related to planning and execution of training sessions (Table 6). Differences in session models across sports are mainly explained by competition-specific demands, seasonal considerations, logistic factors, movement constraints for the modality, and associated load-tolerance considerations.

**Table 6:** Sport-specific features related to planning and execution of training sessions

Sport	Specific features
Biathlon	Biathletes perform considerable amounts of cross-training in the form of cycling and running, particularly during the preparation period when access to snow is limited The intensity demands during competitions vary according to terrain variations, and biathletes apply sub-techniques optimized for different speed-incline combinations. Hence, most (roller) ski-interval sessions are conducted in competition-specific terrain with combinations of intensity zones Z1 sessions are mainly conducted in the lower part of the zone, particularly during running and cycling sessions. However, long-slow distance sessions may drift to Z2 during uphill skiing to ensure proper technique Interval sessions are often performed in combination with shooting during the recovery periods. Five intervals are often applied to ensure equal amount of shooting in the standing and prone position. Here, warmup shots close to resting state are performed in advance for preparation purposes
XC-skiing	XC skiers perform considerable amounts of cross-training (e.g., running with or without poles on soft terrain during the preparation period where access to snow is limited) The intensity demands during competitions vary according to terrain variations, and XC-skiers apply sub-techniques optimized for different speed-incline combinations. Hence, most (roller) ski-interval sessions are conducted in competition-specific terrain with combinations of intensity zones Z1 sessions are mainly conducted in the lower part of the zone, particularly during running sessions. However, long-slow distance sessions may drift to Z2 during uphill skiing to ensure proper technique
L-D running	Long-distance runners generally apply shorter session models than other endurance sports (particularly Z1–3), and this is mainly explained by the high mechanical loading demands Much of the accumulated running kilometres in Z1–3 sessions is undertaken with cushioned shoes on forgiving surfaces (dirt roads/forest paths) instead of paved roads to reduce mechanical loading and maximize training volume. The higher the intensity, and the closer to the competition season, the more running sessions are undertaken on rubberized track with spike shoes Cross-training sessions are mainly applied as alternative training during injury rehabilitation periods, but some athletes perform cross-training sessions to cope with the high total training volume during the preparation period Treadmill running sessions are preferred when weather/winter conditions are poor (rain, snow, ice) and for intensity control (particularly during Z3 sessions) Z1 sessions are mainly conducted in the upper part of the zone, and sometimes in Z2, to ensure proper running technique
Road cycling	Because of the long competition duration, and the notion that cycling is a gentle locomotion modality with low injury risk (disregarding falls and crashes), most sessions are in the range 3–6 h Most intensive sessions are performed as competitions (most road cyclists compete 50–70 days per year). However, each race is characterized by a broad range of intensities, depending on the type of competition (mass starts versus time trials, single-day races versus. stage races, hilly versus flat terrain, etc.) and role within the team (captain versus domestiques, climbers versus sprinters, etc.) Most of the remaining intensive training (beside competitions) is integrated in LIT sessions (e.g., cycling uphill or cycling with reduced drag)
Rowing	Considerable amounts of cross-training sessions in the form of cycling (summer) or XC-skiing (winter) are performed to achieve sufficient total training volume. Moreover, warmups and cool downs in conjunction with (ergometer) rowing interval sessions involve cycling or XC-skiing, depending on season Speed training is integrated in rowing-specific long-slow distance or interval sessions 2–3 times per week to improve acceleration or top speed
Speed skating	Because an effective skating position is muscularly demanding over time (inducing local fatigue, back pain, etc.), long skating sessions are challenging to perform. Moreover, the restricted access to ice in Norway during the summer season further reduces the number of skating-specific sessions. Therefore, most low- and moderate-intensity sessions (Z1-3) consist of road cycling, while most high-intensive sessions are performed on skates. The cycling training increases the tolerance for more frequent, intensive, or longer skating sessions. Cycling is also preferred during warmups and cool down in conjunction with intensive skating sessions to fully utilize the limited ice access and to provide an aerobic stimulus Speed-skating intervals (Z4) are typically performed in small groups (mostly two to four but sometimes eight to ten athletes) to reduce air drag and thereby increase the speed. However, competition-specific speed training (mainly Z5) is normally practiced individually for intensity control purposes Because speed-skating is muscularly demanding, longer interval recoveries are applied in speed-skating sessions compared to corresponding sessions in other locomotion modalities and sports
Swimming	Interval training is performed across all intensity zones, and continuous work is rarely applied. However, the interval recoveries in Z1-2 sessions are very short, so such sessions bare great resemblances to continuous, long-slow distance sessions. The application of low-intensive interval sessions is mainly for nutrition/fuelling purposes, providing technical feedback, and taking lactate measurements Crawl is the main stroke for long-distance swimmers during practically all moderate- to high-intensive sessions. Other strokes may be used during Z1-2 work, but never constituting more than 30–40% of the total swimming time. The closer to the competition season, the higher the proportion of crawl swimming Special equipment (e.g., boards, zoomers and paddles) is sometimes used during Z1-2 sessions for technical development purposes, to increase stroke power output, or to reduce the load during low-intensive swimming Separate Z1-2 leg sets are sometimes conducted for technical development purposes or development of aerobic capacity Running is occasionally used at cross-training in Z1 and Z3 sessions during the preparation period, but also as warmup to swimming sessions To avoid logistic challenges in crowded pools, swimmers apply “slot times” instead of recovery times (e.g., athlete 1 starts at 0:00, 1:00, 2:00, etc., while athlete 2 starts at 0:15, 1:15, 2:15 min) “Broken” is a common method for Z5-intervals. Here, the competition discipline (e.g., 800 or 1500 m) is broken down to shorter intervals (e.g., 100 m). The aim is to swim the intervals at race pace with short recovery periods in between. The closer to the competition season, the shorter the recovery periods
Triathlon	Because triathlon consists of swimming, road cycling and running, combined sessions (so called brick workouts) are frequently applied to manage modality transitions. Swimming/cycling or cycling/running are combined for physiological adaptation purposes and efficient change of equipment, footwear and outfit. Moreover, athletes try out fuel/nutrition intake during such sessions to optimize individual competition routines The cycling part of the triathlon competition consists of many tight turns, accompanied with decelerations and subsequent accelerations. To cope with these demands, cycling intervals are implemented with brief (5–10 s) sprints every 1–2 min to simulate the competition-specific situation It is considered crucial to be up near the front in the initial swimming part of the triathlon competitions, as it costs a lot of energy to pass competitors in a crowded open-water swim. Hence, elite triathletes practice fast starts in many swimming interval sessions. For example, the first 25 m of each 100 m interval are at considerably faster than race pace, while the remaining 75 m of each interval are conducted at race pace Brick workouts are designed to simulate competition-specific demands. Because the competition-specific intensity is between Z3 and 4, most brick sessions are performed at the same intensity. Hence, brick sessions in Z3 and 4 do not differ considerably

## Discussion

This is the first study to describe and compare training session models across intensities and endurance sports. The duration of LIT sessions varies substantially across sports, ranging from 30 min to 7 h, mainly due to modality-specific constraints and load tolerance considerations, while MIT and HIT sessions differ less across sports and are mainly conducted as intervals (or competitions) in specific modalities. Overall, both MIT and HIT interval sessions are characterized by a high AWD, a progressive increase in intensity throughout the session, and a controlled rather than exhaustive execution approach. In the following paragraphs, we will discuss the quantitative and qualitative aspects of these session models and potential underlying mechanisms in more detail.

This study clearly demonstrates that LIT is the most prescribed type of training session in elite endurance sport, in line with previous studies based on quantification of training performed [2, 7, 13, 14, 18, 22, 38]. Most LIT sessions are prescribed and executed in Z1, interspersed with Z2 once-to-twice per week, or as part of progressive Z1-sessions. This distinct feature would not have been detected by the commonly applied three-zone scale (LIT, MIT, and HIT), emphasizing the advantage of a more categorized scale (e.g., a six-zone scale as in this study). In this context, it is important to emphasize that elite endurance athletes have a broad intensity range below the first lactate turn point compared with recreational and moderately trained performers. This makes the potential range of intensity and duration combinations within LIT larger for elite athletes and a more important programming consideration for their coaches. One might speculate that Z2 training costs too much for elite endurance athletes, making them less recovered and poorer prepared for the subsequent intensive sessions. According to several of the present coaches, Z2 training is mainly implemented for technical reasons, as an effective force signature in the movement cycle sometimes requires a minimum speed or power output. Some of the analyzed sports perform LIT as long intervals to provide short intermissions for nutrition/fueling, technical feedback, and lactate measurements. However, since the latter intervals are quite long and the intermittent recoveries are relatively short, such sessions practically act as continuous LIT sessions from a perceptual and physiological perspective. Similarly, terrain variations in sports such as biathlon and XC-skiing make LIT sessions more stochastic [39, 40]. The prevailing notion is that most LIT sessions must be sufficiently easy to ensure that the subsequent hard sessions can be conducted with sufficient quality. LIT sessions have misguidedly been termed “recovery workouts” by several practitioners over the years [22], suggesting that these sessions do not elicit adaptations themselves but rather “accelerate” recovery prior to the next hard session. We argue that this interpretation is erroneous for two important reasons. First, the concept of any form of recovery acceleration from an intervening workout lacks support in the scientific literature, although the “low” load of such sessions likely causes limited interference with the ongoing recovery process. Second, frequent and voluminous LIT is considered an important stimulus for inducing peripheral aerobic adaptations [41] and improving work economy [42, 43]. At least three adaptive signaling pathways (through which exercise of different intensities and durations can impact protein composition of working muscle over time) have well-demonstrated signaling roles, mediated through specific kinases, and aggregated by the PGC1a gene [44]. The pathways triggered by high energy phosphate depletion (i.e., large reductions in ATP/AMP ratio) and by elevated production of reactive oxygen and nitrogen species both show rapidly evolving feedback inhibition of signal amplitude as signal mediated adaptations occur [45–47]. That is, adaptive feedback inhibition reduces the adaptive return from these pathways with repeated HIT bouts over time. In contrast, it appears that elevated intracellular calcium concentration associated with the excitation-contraction coupling process remain responsive across longer training time frames due to greater potential for modulation via exercise duration × intensity interaction, with essentially no feedback inhibition of the primary signal within and across motor units. Accordingly, it may seem that years of accumulated wisdom among elite coaches is consistent with how different signaling pathways coalesce to determine the overall adaptive enrichment of the endurance phenotype.

Notably, AWD for LIT sessions varies markedly, both within and across sports. Within-sport differences are mainly explained by session purpose (i.e., extra-long versus short, long-slow distance), while between-sport differences were explained by competition-specific demands, movement constraints for the modality and associated load-tolerance considerations. The latter aspect is in line with Sandbakk et al. [30], who recently developed a theoretical framework for the impact of physiological and biomechanical mechanisms associated with different locomotion modalities on training load management in endurance exercise. According to their theory, the combination of weight-bearing exercise and rapid plyometric power production in long-distance running puts high loads on muscles and tendons during each step, likely explaining why the duration of LIT sessions in long-distance running is relatively low compared to most other endurance sports. However, elite runners seem to compensate for this “low” volume by training twice a day and performing some of the LIT sessions in the upper range of Z1, sometimes approaching Z2 [22].

Speed skating is also muscularly demanding but for other reasons. The small angles in the hip and knee, in addition to the static upper body position and long duty cycle of an effective skating stroke, together induce intermittent blood-flow restrictions in the working muscles [6, 48]. Hence, speed skaters typically prefer cycling instead of the skating-specific modality during LIT and MIT sessions, as well as for warmups and cool downs. Nils van der Poel, double gold medalist in the 2022 Beijing Winter Olympics, followed this approach to the extreme with 6–7 h rides on the bike five times per week during preparation training and considerable amounts of MIT cycling in the subsequent phase [49].

Road cyclists perform longer but fewer training sessions compared with the other sports. The preference for and tolerance of voluminous road cycling sessions can mainly be explained by the concentric only and nonweight bearing loading, the long-duration competition format, and the fact that cyclists draft behind teammates/competitors and coast downhill in substantial parts of the sessions. Careful examination of elite cyclists reveals that 10–20% of all cycling sessions are spent at a power output < 0.75 W/kg [10]. While a runner absorbs a huge mechanical load when running downhill, a cyclist coasting downhill is normally resting the active musculature.

Swimming also involves nonweight-bearing exercise and low contraction velocity movement [30]. Swimmers perform shorter LIT sessions than road cyclists. To obtain a relatively high training volume, these athletes seem to compensate by consistently swimming twice a day, with the first session performed in the early morning. This approach can at least partly be explained by restricted access to swimming halls in the middle of the day, as school swimming is generally prioritized by local authorities. Interestingly, in contrast to their high-volume training, most swimming events are dramatically shorter in duration compared with road cycling and most other traditional endurance sport events.

Rowing also involves nonweight-bearing and low contraction velocity movement, but the injury risk of overloaded back and ribs, particularly with modern “cleaver” rowing blades, has led rowers to implement a larger proportion of LIT as cross-training [30]. In XC skiing and biathlon, the athletes distribute training time across varying sub-techniques while skiing on snow or using roller skis [7, 8, 25, 26]. However, the best athletes do not perform longer LIT sessions than cyclists, rowers, or swimmers. This can be explained by the moderately high muscular loads of skiing uphill, in addition to the strong focus on and accompanying strain associated with maintaining effective technique (and appropriate switching between multiple subtechniques) in complex movements [30].

This study shows that many of the best practitioners within endurance sports supplement their LIT sessions in the specific modalities with cross-training, in line with previous studies [1, 7, 8, 25]. The application of cross-training differs substantially across sports, not only for movement constraints and associated load management but also for seasonal reasons. Because of the limited access to snow during the summer, XC skiers and biathletes perform many running and cycling sessions, respectively. Likewise, rowers execute numerous land-based sessions as running, cycling, or XC skiing (perhaps a distinctly Norwegian cross-training modality) during the winter. Other supporting arguments for cross-training in research literature include injury prevention, general central capacity effects and prevention of training monotony [50, 51]. A plausible question within this context is whether long-distance runners should compensate for their “low” volume (compared with the other analyzed sports) by adding more cross-training sessions to maximize the training stimulus with lower muscular-mechanical load. However, a common notion among the interviewed coaches was that cross-training modality must bear sufficient physiological and mechanical resemblances to the specific demands to maximize the odds for positive adaptations (Table 5), in line with the principle of specificity [52]. Alternative locomotion modalities for runners (e.g., cycling and XC skiing) are less used (in most cases limited to injury rehabilitation processes) and may be too removed from the specific demands, increasing the odds for maladaptations. Running is also unique among endurance sports in that cycle frequency/cadence does not and cannot be manipulated very much across a broad range of intensities/speeds. More specifically, the cadence may only increase 10% from LIT to HIT for a distance runner. For a rower or kayak paddler, cadence can vary at least twofold from Z1 to Z5, with the force signature maintained relatively stable. These issues may partly explain why cross-training in long-distance running mainly is restricted to injury rehabilitation processes. Overall, the underlying mechanism of cross-training remains poorly understood, and future longitudinal studies should aim to explore the training transfer efficiency of varying types of cross-training.

Based on the large variations in LIT session duration across the analyzed sports in this study, it is reasonable to question well-established training load assessment tools such as training impulse (TRIMP) and session rating of perceived exertion (session RPE). While these concepts only take training volume and intensity into account [53–55], it seems clear that the choice of exercise modality influences effort beyond commonly applied external and internal load measurements. We argue that these methods are not valid for comparisons of training load across exercise modalities, for example, by comparing sports or when comparing the load across different modalities. Foster et al. [56] have also indicated that session RPE is mode dependent, but more studies are warranted to verify this feature.

Intensive sessions in the form of MIT and HIT are considered fundamental for performance progression by all the participating coaches in this study, and the planning and implementation of training are mainly centered around such key sessions. Most MIT sessions are performed as intervals, although several sports also apply continuous work. Competitions account for parts of MIT or as elements of LIT, and the stochasticity of many competition formats such as cycling and XC skiing results in some intensity undulation. A specific feature for triathlon is the application of combined modalities (so-called brick workouts), where swimming/cycling or cycling/running are frequently applied during MIT sessions to manage modality transitions. Overall, the analyzed sports implement considerably more MIT than HIT sessions across the annual cycle. This strategy has been a part of the training philosophy in several Norwegian endurance sports over the last two decades [57]. Here, a fundamental feature is the application of double threshold sessions (i.e., both morning and afternoon) twice a week, with blood lactate concentrations in the range 2–4.5 mmol/L. Marius Bakken, a former Norwegian 5000 m record holder (13:06 min) is considered the originator of this concept, and he has argued that Z3 intervals (particularly microintervals lasting only 45–60 s) allow for accumulation of work at faster and more race relevant running speeds than continuous training in the same lactate-based zone, without the negative consequences of HIT in the form of fatigue and subsequent recovery [57]. Half of the coaches in this study and numerous elite coaches worldwide have adopted double threshold sessions in their weekly preparation training, representing a novelty in the current training of elite endurance runners.

The HIT sessions presented in this study are mainly conducted as intervals, although competitions constitute a substantial part of most sports. In road cycling, elements of HIT are conducted during LIT sessions. In triathlon, Z4 sessions are often conducted as brick workouts. Overall, a common and logical trend across all sports is that interval times and accumulated working duration for interval sessions decrease with increasing intensity. Recovery time between intervals depends on interval time and intensity, but we observed a clear trend towards lower work-to-rest ratio with increasing intensity. The variations in MIT and HIT session design across sports can mainly be explained by corresponding movement constraints and load management considerations as explained previously for LIT sessions, although the differences between sports diminish with increasing training intensity.

Ever since the first studies on interval training were published in the 1960s [58, 59], a plethora of research has been devoted to this topic. Interestingly, the best practice interval sessions described in the present paper differ considerably from most of the models tested in previous intervention studies that are the building blocks of current established scientific recommendations [20, 31, 60]. First, our analyzed sports perform interval sessions across a considerably wider intensity range compared with research literature. Comprehensive and highly cited review papers recommend athletes to reach at least 90% of their maximal oxygen uptake during interval sessions (or ≥ 95% of the minimal velocity/power that elicits maximal oxygen uptake) to elicit both maximal cardiovascular and peripheral adaptations [20, 31, 60]. Secondly, AWD for interval sessions is also considerably lower in most scientific studies [31, 60] than those presented here. Fundamentally, elite coaches use AWD to adjust both stimulus and progression “between” intensity adjustments (stair-step model) far more than most recreational athletes and researchers, who emphasize mainly intensity as a “lever” for managing the HIT prescription. Third, the observed trend towards lower work-to-rest ratio with increasing intensity has not previously been established in scientific studies. However, the predominant application of passive recoveries is in line with recommendations from research literature, as active recoveries can lower muscle oxygenation, impair phosphocreatine resynthesis and, thereby, trigger anaerobic system engagement during the following effort [31].

Another notable finding from this study is that very few interval sessions are performed to the point of power or pace “failure.” Instead, these sessions are characterized by an even pacing across bouts or even a small but progressive increase in intensity (crossing through, for example, upper Z3 to upper Z4), a semiexhausting effort and high AWD. Importantly, maintaining good technique (i.e., avoiding technical collapse and “floundering” near the end of work bouts) is emphasized. Some intervention studies have applied intervals with maximal sustainable work intensity, aiming to achieve the highest possible average speed or power (so called “maximum session effort” or isoeffort approach) [61–63]. The interviewed coaches argue that such an all-out approach is not sustainable over time for several reasons. In a short-term perspective, an all-out session execution approach can lead to an undesired and poorly timed peaking response (provided that the recoveries between such hard sessions are sufficient). In a long-time perspective, an all-out approach limits the accumulated load of MIT and HIT due to shorter work time in single sessions and longer recovery time after sessions. Concurrently, this increases the odds for overtraining and burnout due to the physical and mental strain associated with such sessions. The best practitioners are, therefore, especially cautious not to overuse all-out intensive sessions or introduce them too early in the annual cycle [7, 16, 22, 25, 35], a notion in line with traditional periodization thinking [64]. Alternatively, controlled and semiexhausting interval sessions may effectively stimulate adaptation through the interaction between high intensity and larger accumulated work that can be achieved before the onset of fatigue, compared with an all-out approach [61, 65–67].

All the key informant coaches in this study consider training quality highly important for performance development. Here, “quality” is not synonymous with “intensity” as often seen in popular science literature. Instead, training quality is defined as the degree of excellence related to how the training sessions are executed to optimize adaptations and/or improve overall performance [33, 69]. This includes the ability to optimize processes that affect the execution of training sessions in relation to the intended purpose. Intensity discipline in relation to the training prescription is an example of this quality emphasis observed at the elite level. Training quality can be developed and fine-tuned over time through optimal application of monitoring tools and good communication among the athlete, coach, and supporting staff. Obtained information related to readiness, exercise load, and recovery state form a basis for subsequent decision making [69], and this is continuously subject to improvement through a circular learning process where planning, execution and debriefing/evaluation are the fundamental stages [33, 68]. The present coaches describe a culture of continuous learning and development through constructive interactions with the athletes.

Although this study has described a variety of session models across sports, the best practitioners tend to apply a limited set of session models within each zone. In this way, each key session acts as a test where heart rate, blood lactate concentration, speed/power output, and perceived fatigue/exertion can be compared from week to week. The principle of control is a fundamental feature of elite sport to determine whether athletes adapt to the training, identify individual responses, monitor fatigue and accompanying need for recovery, and minimize the probability of nonfunctional overreaching, illness and injury [55, 70]. It is also reasonable to assume that implementation of well-known sessions increases the likelihood for increased training quality. Interestingly, all coaches have primarily adjusted their training session models to the individual athlete and sport-specific demands, rather than based solely on sex, as previously described more generally [71].

A distinct feature across all the analyzed sports in this study is the alternating rhythmicity of hard and easy workouts. The legendary track and field coach Bill Bowerman popularized this concept in the 1960s [22]. This was also a fundamental feature of Matveyev’s traditional periodization model founded at the same time [64], with strong links to the principle of stimulus and response (also known as the overcompensation or training adaptation principle). That is, training stress leads to acute fatigue and damage to physiological structures, and during the subsequent restitution phase, the organism does not only return to the original condition but overcompensates to be better prepared for the next stress. The larger the training stress, the longer the restitution time required [72]. Importantly, many of the coaches regard the training day (not only each session) as the unit of stress being managed. Therefore, amplifying the intensive load during a planned “high stress” training day is more sustainable than adding an additional high stress training day to the microcycle. MIT and HIT both induce high stress, particularly given the high absolute intensities and associated metabolic flux of elite performers, combined with the AWD that is prescribed and executed. The present study, together with other recently published studies, shows that consecutive hard training days rarely occur and that the hard–easy rhythmicity also holds true for today’s elite endurance practitioners [7, 16, 22, 25, 35]. Hard and easy sessions seem to stimulate a complex set of overlapping and complementary adaptations [73, 74], justifying the systematic training intensity variation for performance development in endurance sports. Overall, we would argue that elite coaches use this day-to-day rhythmicity to carefully manage and, to a substantial extent, “polarize” training stress, not work intensity, to ensure that recovery is achieved. Elite coaching is about managing the systemic cost of maintaining a high training frequency and volume, and thereby a high adaptive signal. Across sports, the success of these elite coaches is quantified in terms of long-term thinking and “staying healthy and being able to do the work required for success.”

Some study limitations should be acknowledged. First, it is likely that the present results are influenced by a Norwegian “group culture” bias, and other roads may also lead to Rome. Although the key informants in this study have coached numerous world-leading athletes, they have also applied the same training system to several other less successful athletes. Moreover, the intensity scale outlined here (Table 2) has been used by Norwegian elite endurance athletes over the last two decades. Previous studies have presented several arguments to explain why standardized intensity zone systems are imperfect tools [2, 16, 22, 37]. Slight inconsistencies in AWD within the same zone can be observed when comparing present findings with previous studies [16, 22]. Inconsistencies across studies are expected because (1) the intensive zones are “narrow” (i.e., small differences in heart rate, blood lactate and RPE), and (2) MIT/HIT sessions tend to overlap intensity zones. Although intensity scales can be criticized for several reasons, we argue that the potential error sources are outweighed by the improved communication among practitioners that a common scale facilitates.

Finally, we do not believe Norwegian athletes are “physiologically biased” towards a genotype that is uniquely responsive to the training characteristics described here. More likely, the Norwegian endurance sport success is grounded on a culture which includes an appreciation for “endurance.” Norwegian champions often describe an active childhood that included lots of hiking, skiing, cycling, etc., just as a function of living. So, relative to the size of the country, we could argue that a large fraction of Norwegian children has good local conditions for (1) sampling a variety of endurance sports and (2) meeting local coaches with a good understanding of the endurance training process.

## Conclusions

The unique training session templates presented here are derived from world-leading coaches, whose athletes have won more than 350 medals in international championships. Overall, large variations in session loading factors were observed across sports, although the differences diminish with increasing intensity. AWD for LIT sessions ranges from approximately 30 min to 7 h, with differences being mainly explained by modality-specific constraints and accompanying consequences for load tolerance. For the same reasons, in addition to seasonal considerations, several sports perform large amounts of LIT using cross training. Intensive sessions (MIT and HIT) are considered paramount for performance progression by all coaches, and all sports perform considerably more MIT than HIT sessions across the annual cycle. Although most intensive sessions are conducted as intervals, competitions also account for a large proportion. Best practice interval sessions are characterized by a controlled, nonall-out approach, high AWD, and a slight progressive increase in intensity throughout. We also observed a trend towards lower work-to-rest ratio with increasing intensity.

## Declarations

### Availability of Data and Materials

All data and materials support the published claims and comply with field standards. To protect the anonymity of the key informants, as well as their athletes, the transcribed interviews cannot be made publicly available.

## References

[1] Fiskerstrand A, Seiler KS. Training and performance characteristics among Norwegian international rowers 1970–2001. Scand J Med Sci Sports. 2004;14:303–10. 15387804 10.1046/j.1600-0838.2003.370.x

[2] Seiler S, Tønnessen E. Intervals, thresholds, and long slow distance: the role of intensity and duration in endurance training. Sportscience. 2009;13:32–53.

[3] Guellich A, Seiler S, Emrich E. Training methods and intensity distribution of young world-class rowers. Int J Sports Physiol Perform. 2009;4:448–60. 20029096 10.1123/ijspp.4.4.448

[4] Bourgois J, Steyaert A, Boone J. Physiological and anthropometric progression in an international oarsman: a 15-year case study. Int J Sports Physiol Perform. 2014;9:723–6. 24085306 10.1123/ijspp.2013-0267

[5] Steinacker JM, Lormes W, Lehmann M, Altenburg D. Training of rowers before world championships. Med Sci Sports Exerc. 1998;30:1158–63. 9662689 10.1097/00005768-199807000-00022

[6] Orie J, Hofman N, de Koning JJ, Foster C. Thirty-eight years of training distribution in Olympic speed skaters. Int J Sports Physiol Perform. 2014;9:93–9. 24408352 10.1123/IJSPP.2013-0427

[7] Tønnessen E, Sylta Ø, Haugen T, Hem E, Svendsen I, Seiler S. The road to gold: Training and peaking characteristics in the year prior to a gold medal endurance performance. PLoS ONE. 2014;9: e101796. 25019608 10.1371/journal.pone.0101796 PMC4096917

[8] Sandbakk Ø, Holmberg HC. Physiological capacity and training routines of elite cross-country skiers: Approaching the upper limits of human endurance. Int J Sports Physiol Perform. 2017;12:1003–11. 28095083 10.1123/ijspp.2016-0749

[9] Svendsen IS, Tønnesen E, Tjelta LI, Ørn S. Training, performance, and physiological predictors of a successful elite senior career in junior competitive road cyclists. Int J Sports Physiol Perform. 2018;13:1287–92. 29745739 10.1123/ijspp.2017-0824

[10] van Erp T, Sanders D, de Koning JJ. Training characteristics of male and female professional road cyclists: a 4-year retrospective analysis. Int J Sports Physiol Perform. 2020;4:534–40. 10.1123/ijspp.2019-0320 31722298

[11] Gallo G, Mateo-March M, Gotti D, Faelli E, Ruggeri P, Codella R, Filipas L. How do world class top 5 Giro d’Italia finishers train? A qualitative multiple case study. Scand J Med Sci Sports. 2022;32:1738–46. 35686390 10.1111/sms.14201 PMC9796663

[12] Gallo G, Mateo-March M, Gotti D, Maunder E, Codella R, Ruggeri P, Faelli E, Filipas L. The weekly periodization of top 5 Tour de France general classification finishers: a multiple case study. Int J Sports Physiol Perform. 2023;18:1313–20. 37709277 10.1123/ijspp.2023-0142

[13] Casado A, Hanley B, Santos-Concejero J, Ruiz-Pérez LM. World-class long-distance running performances are best predicted by volume of easy runs and deliberate practice of short-interval and tempo runs. J Strength Cond Res. 2021;35:2525–31. 31045681 10.1519/JSC.0000000000003176

[14] Casado A, González-Mohíno F, González-Ravé JM, Foster C. Training periodization, methods, intensity distribution, and volume in highly trained and elite distance runners: A systematic review. Int J Sports Physiol Perform. 2022;17:820–33. 35418513 10.1123/ijspp.2021-0435

[15] Tjelta LI. The training of international level distance runners. Int J Sports Sci Coach. 2016;11:122–34.

[16] Haugen T, Sandbakk Ø, Enoksen E, Seiler S, Tønnessen E. Crossing the golden divide: the science and practice of training world-class 800- and 1500-m runners. Sports Med. 2021;51:1835–54. 34021488 10.1007/s40279-021-01481-2 PMC8363530

[17] Hellard P, Avalos-Fernandes M, Lefort G, Pla R, Mujika I, Toussaint JF, Pyne DB. Elite swimmers’ training patterns in the 25 weeks prior to their season’s best performances: Insights into periodization from a 20-years cohort. Front Physiol. 2019;10:363. 31031631 10.3389/fphys.2019.00363 PMC6470949

[18] González-Ravé JM, Hermosilla F, González-Mohíno F, Casado A, Pyne DB. Training intensity distribution, training volume, and periodization models in elite swimmers: a systematic review. Int J Sports Physiol Perform. 2021;16:913–26. 33952709 10.1123/ijspp.2020-0906

[19] Pollock S, Gaoua N, Johnston MJ, Cooke K, Girard O, Mileva KN. Training regimes and recovery monitoring practices of elite British swimmers. J Sports Sci Med. 2019;18:577–85. 31427881 PMC6683628

[20] Billat V. Interval training for performance: a scientific and empirical practice: special recommendations for middle- and long-distance running. Part I: aerobic interval training. Sports Med. 2001;1:13–31. 10.2165/00007256-200131010-00002 11219499

[21] Kenneally M, Casado A, Gomez-Ezeiza J, Santos-Concejero J. Training intensity distribution analysis by race pace vs. physiological approach in world-class middle- and long-distance runners. Eur J Sport Sci. 2021;21:819–26. 32449500 10.1080/17461391.2020.1773934

[22] Haugen T, Sandbakk Ø, Seiler S, Tønnessen E. The training and development of world-class long-distance running performance: an integration of scientific and best practice literature. Sports Med Open. 2022;8:46. 35362850 10.1186/s40798-022-00438-7 PMC8975965

[23] Enoksen E, Tjelta AR, Tjelta LI. Distribution of training volume and intensity of elite male and female track and marathon runners. Int J Sports Sci Coach. 2011;6:273–93.

[24] Torvik PØ, Solli GS, Sandbakk Ø. The training characteristics of world-class male long-distance cross-country skiers. Front Sports Act Living. 2021;3: 641389. 33718870 10.3389/fspor.2021.641389 PMC7947281

[25] Solli GS, Tønnessen E, Sandbakk Ø. The training characteristics of the world’s most successful female cross-country skier. Front Physiol. 2017;8:1069. 29326603 10.3389/fphys.2017.01069 PMC5741652

[26] Sandbakk Ø, Holmberg HC. A reappraisal of success factors for Olympic cross-country skiing. Int J Sports Physiol Perform. 2014;9:117–21. 24088346 10.1123/ijspp.2013-0373

[27] Mujika I. Olympic preparation of a world-class female triathlete. Int J Sports Physiol Perform. 2014;9:727–31. 24088819 10.1123/ijspp.2013-0245

[28] Cejuela R, Sellés-Pérez S. Road to Tokyo 2020 Olympic Games: Training characteristics of a world class male triathlete. Front Physiol. 2022;13: 835705. 35514361 10.3389/fphys.2022.835705 PMC9065268

[29] Cejuela R, Selles-Perez S. Training characteristics and performance of two male elite short-distance triathletes: From junior to “world-class.” Scand J Med Sci Sports. 2023;33:2444–56. 37632141 10.1111/sms.14474

[30] Sandbakk Ø, Haugen T, Ettema G. The influence of exercise modality on training load management. Int J Sports Physiol Perf. 2021;16:605–8. 10.1123/ijspp.2021-0022 33639611

[31] Buchheit M, Laursen PB. High-intensity interval training, solutions to the programming puzzle: Part I: cardiopulmonary emphasis. Sports Med. 2013;43:313–38. 23539308 10.1007/s40279-013-0029-x

[32] Haugen T. Best practice coaches: an untapped resource in sport science research. Int J Sports Physiol Perf. 2021;16:1215–6. 10.1123/ijspp.2021-0277 34271549

[33] Bucher Sandbakk S, Walther J, Solli GS, Tønnessen E, Haugen T. Training quality—what is it and how can we improve it? Invited commentary. Int J Sports Physiol Perform. 2023;18:557–60. 36965489 10.1123/ijspp.2022-0484

[34] Greatest Sporting Nation. The quest for the best. Retrieved from https://greatestsportingnation.com/. Accessed 1 Dec 2023.

[35] Tønnessen E, Svendsen I, Rønnestad B, Hisdal J, Haugen T, Seiler S. The annual training periodization of 8 World Champions in orienteering. Int J Sports Physiol Perform. 2015;10:29–38. 24896267 10.1123/ijspp.2014-0005

[36] Sylta O, Tønnessen E, Seiler S. From heart-rate data to training quantification: a comparison of 3 methods of training-intensity analysis. Int J Sports Physiol Perform. 2014;9:100–7. 24408353 10.1123/IJSPP.2013-0298

[37] Seiler S. What is best practice for training intensity and duration distribution in endurance athletes? Int J Sports Physiol Perform. 2010;5:276–91. 20861519 10.1123/ijspp.5.3.276

[38] Casado A, Hanley B, Ruiz-Pérez LM. Deliberate practice in training differentiates the best Kenyan and Spanish long-distance runners. Eur J Sport Sci. 2020;20:887–95. 31724902 10.1080/17461391.2019.1694077

[39] Noordhof DA, Danielsson ML, Skovereng K, Danielsen J, Seeberg TM, Haugnes P, Kocbach J, Ettema G, Sandbakk ØB. The dynamics of the anaerobic energy contribution during a simulated mass-start competition while roller-ski skating on a treadmill. Front Sports Act Living. 2021;3: 695052. 34308347 10.3389/fspor.2021.695052 PMC8297164

[40] Haugnes P, Kocbach J, Luchsinger H, Ettema G, Sandbakk Ø. The interval-based physiological and mechanical demands of cross-country ski training. Int J Sports Physiol Perform. 2019;14:1371–7. 30958055 10.1123/ijspp.2018-1007

[41] Bishop D, Botella J, Grantha C. CrossTalk opposing view: exercise training volume is more important than training intensity to promote increases in mitochondrial content. J Physiol. 2019;597:4115–8. 31309570 10.1113/JP277634

[42] Morgan DW, Bransford DR, Costill DL, Daniels JT, Howley ET, Krahenbuhl GS. Variation in the aerobic demand of running among trained and untrained subjects. Med Sci Sports Exerc. 1995;27:404–9. 7752868

[43] Nelson RC, Gregor RJ. Biomechanics of distance running: a longitudinal study. Res Q. 1976;47:417–28. 1069331

[44] Hoppeler H. Molecular networks in skeletal muscle plasticity. J Exp Biol. 2016;219:205–13. 26792332 10.1242/jeb.128207

[45] Granata C, Oliveira RSF, Little JP, Bishop DJ. Forty high-intensity interval training sessions blunt exercise-induced changes in the nuclear protein content of PGC-1α and p53 in human skeletal muscle. Am J Physiol Endocrinol Metab. 2020;318:E224–36. 31794264 10.1152/ajpendo.00233.2019 PMC7052577

[46] Granata C, Jamnick NA, Bishop DJ. Principles of exercise prescription, and how they influence exercise-induced changes of transcription factors and other regulators of mitochondrial biogenesis. Sports Med. 2018;48:1541–59. 29675670 10.1007/s40279-018-0894-4

[47] McConell GK, Lee-Young RS, Chen ZP, Stepto NK, Huynh NN, Stephens TJ, Canny BJ, Kemp BE. Short-term exercise training in humans reduces AMPK signalling during prolonged exercise independent of muscle glycogen. J Physiol. 2005;568:665–76. 16051629 10.1113/jphysiol.2005.089839 PMC1474728

[48] Foster C, Rundell KW, Snyder AC, Stray-Gundersen J, Kemkers G, Thometz N, Broker J, Knapp E. Evidence for restricted muscle blood flow during speed skating. Med Sci Sports Exerc. 1999;31:1433–40. 10527316 10.1097/00005768-199910000-00012

[49] van der Poel N. How to skate a 10k. Retrieved from https://www.howtoskate.se/_files/ugd/e11bfe_b783631375f543248e271f440bcd45c5.pdf. Accessed 1 Dec 2023.

[50] Loy SF, Hoffmann JJ, Holland GJ. Benefits and practical use of cross-training in sports. Sports Med. 1995;19:1–8. 7740244 10.2165/00007256-199519010-00001

[51] Foster C, Hector LL, Welsh R, Schrager M, Green MA, Snyder AC. Effects of specific versus cross-training on running performance. Eur J Appl Physiol Occup Physiol. 1995;70:367–72. 7649149 10.1007/BF00865035

[52] Sale D, MacDougall D. Specificity in strength training: a review for the coach and athlete. Can J Appl Sport Sci. 1981;6:87–92. 7016357

[53] Banister EW. Modeling elite athletic performance. In: MacDougall JD, Wenger HA, Green HJ, editors. Physiological testing of the high performance athlete. 2nd ed. Champaign: Human Kinetics Books; 1991. p. 403–24.

[54] Foster C, Daines E, Hector L, Snyder AC, Welsh R. Athletic performance in relation to training load. Wis Med J. 1996;95:370–4. 8693756

[55] Foster C, Rodriguez-Marroyo JA, de Koning JJ. Monitoring training loads: the past, the present, and the future. Int J Sports Physiol Perform. 2017;12:22–8. 10.1123/ijspp.2016-0388 28253038

[56] Foster C, Boullosa D, McGuigan M, Fusco A, Cortis C, Arney BE, Orton B, Dodge C, Jaime S, Radtke K, van Erp T, de Koning JJ, Bok D, Rodriguez-Marroyo JA, Porcari JP. 25 years of session rating of perceived exertion: Historical perspective and development. Int J Sports Physiol Perform. 2021;16:612–21. 33508782 10.1123/ijspp.2020-0599

[57] Casado A, Foster C, Bakken M, Tjelta LI. Does lactate-guided threshold interval training within a high-volume low-intensity approach represent the “next step” in the evolution of distance running training? Int J Environ Res Public Health. 2023;20:3782. 36900796 10.3390/ijerph20053782 PMC10000870

[58] Astrand I, Astrand PO, Christensen EH, Hedman R. Intermittent muscular work. Acta Physiol Scand. 1960;48:448–53. 13794890 10.1111/j.1748-1716.1960.tb01879.x

[59] Christensen EH, Hedman R, Saltin B. Intermittent and continuous running. Acta Physiol Scand. 1960;50:269–78. 14448704 10.1111/j.1748-1716.1960.tb00181.x

[60] Buchheit M, Laursen PB. High-intensity interval training, solutions to the programming puzzle. Part II: anaerobic energy, neuromuscular load and practical applications. Sports Med. 2013;43:927–54. 23832851 10.1007/s40279-013-0066-5

[61] Seiler S, Joranson K, Olesen BV, Hetlelid KJ. Adaptations to aerobic interval training: interactive effects of exercise intensity and total work duration. Scand J Med Sci Sports. 2013;23:74–83. 21812820 10.1111/j.1600-0838.2011.01351.x

[62] Sylta Ø, Tønnessen E, Hammarström D, Danielsen J, Skovereng K, Ravn T, Rønnestad BR, Sandbakk Ø, Seiler S. The effect of different high-intensity periodization models on endurance adaptations. Med Sci Sports Exerc. 2016;48:2165–74. 27300278 10.1249/MSS.0000000000001007

[63] Rønnestad BR, Hansen J, Nygaard H, Lundby C. Superior performance improvements in elite cyclists following short-interval vs effort-matched long-interval training. Scand J Med Sci Sports. 2020;30:849–57. 31977120 10.1111/sms.13627

[64] Matveyev LP. Fundamentals of sport training. Moscow: Progress Publishers; 1981.

[65] Hawley JA, Myburgh KH, Noakes TD, Dennis SC. Training techniques to improve fatigue resistance and enhance endurance performance. J Sports Sci. 1997;15:325–33. 9232558 10.1080/026404197367335

[66] Stepto NK, Hawley JA, Dennis SC, Hopkins WG. Effects of different interval-training programs on cycling time-trial performance. Med Sci Sports Exerc. 1999;31:736–41. 10331896 10.1097/00005768-199905000-00018

[67] Yu M, Stepto NK, Chibalin AV, Fryer LG, Carling D, Krook A, Hawley JA, Zierath JR. Metabolic and mitogenic signal transduction in human skeletal muscle after intense cycling exercise. J Physiol. 2003;546:327–35. 12527721 10.1113/jphysiol.2002.034223 PMC2342514

[68] Haugen T, Tønnessen E, Bucher Sandbakk S, Sandbakk O. Training quality - an unexplored domain within sport science. Int J Sports Physiol Perform. 2023;18:221–2. 36750120 10.1123/ijspp.2022-0500

[69] Boullosa D, Claudino JG, Fernandez-Fernandez J, Bok D, Loturco I, Stults-Kolehmainen M, García-López J, Foster C. The fine-tuning approach for training monitoring. Int J Sports Physiol Perform. 2023;18:1374–9. 37689401 10.1123/ijspp.2023-0154

[70] Bourdon PC, Cardinale M, Murray A, Gastin P, Kellmann M, Varley MC, Gabbett TJ, Coutts AJ, Burgess DJ, Gregson W, Cable NT. Monitoring athlete training loads: Consensus statement. Int J Sports Physiol Perform. 2017;12:2161–70. 10.1123/IJSPP.2017-0208 28463642

[71] Bucher Sandbakk S, Tønnessen E, Haugen T, Sandbakk Ø. Should female and male endurance athletes train or be coached differently on their road to gold? Perceptions among accomplished elite athlete coaches. German J Sports Med. 2022;73:251–7.

[72] Yakovlev NN. Biochemistry of sport in the Soviet Union: beginning, development, and present status. Med Sci Sports. 1975;7:237–47. 1235148

[73] Laursen PB. Training for intense exercise performance: High-intensity or high-volume training? Scand J Med Sci Sports. 2010;20:1–10. 20840557 10.1111/j.1600-0838.2010.01184.x

[74] Talsnes RK, van den Tillaar R, Sandbakk Ø. Effects of increased load of low- versus high-intensity endurance training on performance and physiological adaptations in endurance athletes. Int J Sports Physiol Perf. 2022;17:216–25. 10.1123/ijspp.2021-0190 34611057

